# Supplementary material for: Mental health care in the city of Lubumbashi, Democratic Republic of the Congo: Analysis of demand, supply and operational response capacity of the health district of Tshamilemba
Source: PLoS One. 2023 Apr 5;18(4):e0280089. doi: 10.1371/journal.pone.0280089 (PMC10075459; doi:10.1371/journal.pone.0280089)
Supplement: S1 File — (DOCX) [file pone.0280089.s001.docx]

# Supplementary Text-File 1: Focus Group Discussion-Interview Guide

**Interview Guide - Focus Group**

(English version)

Note to interviewers: The main interview questions are highlighted in **bold**. All *italicized* prompts are optional and should be used variably, if at all, depending on respondent profiles and responses to key questions.

THEME 1: DEMAND FOR MENTAL HEALTH CARE

**1.1 Burden of mental health disorders (problems)**

**What do you think about the situation of mental health disorders (problems) (also called mental illnesses) in your community/health area? Thank you for telling us about it.**

*Types of disorders (including epilepsy) and the manifestations/symptoms most often seen in people with them*

*How do you feel about people with mental health problems? Are you comfortable meeting them, living with them, or do you experience feelings of fear, sadness?*

*How does society view these people with severe mental illnesses (i.e., those who have lost their minds and do not recognize their illnesses)?*

**Tell us about how mental health disorders are a social and public health burden in your family/health area**

*People's beliefs about disorders*

*Public interest in their mental health*

*Contribution to the implementation of mental health activities, psychosocial support and care practices at the community level*

**Do you know anyone in your family/community who is affected by a mental health problem/disorder?**

*Do you meet people in pain on a daily basis?*

*What do you do when you meet them: with your family, on the street...?*

*Which categories of people are affected a lot by these problems/disorders?*

*Are these people wandering in the city of Lubumbashi? Do they adopt anti-social behaviors?*

*Do these people threaten the peace in the family/community?*

*What attitudes does the community have towards these people: punishment, rejection, stigmatization? Please tell us about it.*

**1.2 Care-seeking behavior**

**Take people with mental health problems to health care services or elsewhere for treatment? Why/why not?**

*Will you say that it is out of charity that you bring them to the care?*

*Would you say that it is out of duty to parents/siblings that you bring them to care?*

**Do you see people with mental health problems in health care services?**

*What do you do when you meet them: in the family, on the street, in the hospital...?*

*Is awareness of mental health issues being raised? why? by whom?*

**Before arriving at the health centre_____ or clinic, do people with mental health problems go elsewhere for consultation? Please tell us more.**

*Use of traditional medicine practices*

*Church/prayer group/spiritual counseling attendance*

*Attendance of family and/or traditional leaders*

THEME 2: MENTAL HEALTH CARE SUPPLY

**Can you tell us about the management of mental health disorders at ______ and the private clinics?**

*Is the care effective?*

*If yes, describe what is actually provided as mental health care*

*If not, what are the reasons for the unavailability of care?*

*Where are patients who need mental health care referred?*

*Are there any specialized hospitals in your health zone and/or in the city of Lubumbashi that offer mental health care? Please tell us more.*

*What illnesses are treated in psychiatric hospitals? At what cost?*

**What types of care are currently available for these disorders in your community?**

*Medical care, with psychotropic drugs*

*Psychological care: counseling, psychotherapy, family mediation, etc.*

*Social support for people in difficulty*

*Care by traditional healers*

*Prayer and exorcism sessions by priests and pastors,*

**Can you tell us about how mental health disorders are managed in your community?**

*In health centers - capacity of caregivers to provide this care*

*In hospitals - capacity of caregivers to provide this care*

*In traditional health care facilities - healers' skills*

*In families and communities - attitudes of caregivers*

**What is the content of the minimum package of health activities for primary health care services in your health zone?**

*Curative activities*

*Preventive activities*

*Promotional activities*

*Rehabilitation activities*

*Does this package of activities incorporate mental health care? If yes, what is being done? If no, what are the reasons?*

**Can you tell us about follow-up and referral of mental health cases?**

*Who does the follow-up on mental health cases?*

*Is financial support available in health care services?*

*Can you provide referrals if needed? If so, to whom?*

THEME 3: HEALTH ZONE OPERATIONAL RESPONSE CAPACITY TO ADDRESS MENTAL HEALTH ISSUES

(Reserved for the interview with health professionals)

**Do you think your health area has the technical capacity to address mental health disorders?**

*What medications are often available: psychotropic drugs, neuroleptics including antiepileptic*

*Circuit of supply of these drugs*

**Can you tell us about the situation of psychotropic drugs in your health zone (health center _________, hospitals/clinics, mobile pharmacies)?**

*What medications are often available: psychotropic drugs, neuroleptics including antiepileptic*

*Circuit of supply of these drugs*

*Prices at: a) the central purchasing office of the health zone, b) the health center ____ and clinics/hospitals, c) private pharmacies*

**Can you tell us about the capacity of providers to care for people with mental health problems?**

*Their ability to identify, manage cases and follow up with patients*

*The training they received on mental health*

*Their ability to use the WHO mental health Gap Action Program (mhGAP) if available in the health care setting*

**Can you tell us about the situation of psychotropic drugs in your health zone (health center _________, hospitals/clinics, mobile pharmacies)?**

*What medications are often available: psychotropic drugs, neuroleptics including antiepileptic*

*Circuit of supply of these drugs*

**Can you tell us about how the mental health issue is coordinated in your health zone and in the provincial division of Haut-Katanga**

*Existence of a provincial mental health coordination*

*Existence of a mental health supervisor in the health zone*

*Existence of a mental health referent in primary health care centers*

**Do you have anything to add regarding our interview?**

**What are your expectations regarding mental health issues?**

(These two questions concern all participants)

**Guide d’entretien – Groupe de discussion focalisé**

(Version française)

Note aux enquêteurs : Les principales questions de l’entretien sont surlignées en **gras**. Toutes les relances et invites en *italique* sont facultatives et doivent être utilisées de manière variable, le cas échéant, en fonction de profils des répondants et de leurs réponses aux principales questions.

THEME 1 : DEMANDE DE SOINS DE SANTÉ MENTALE

**1.1 Fardeau des troubles (problèmes) de santé mentale**

**Que pensez-vous de la situation des troubles (problèmes) de santé mentale (qu’on appelle aussi maladies mentales) dans votre communauté/zone de santé ? Merci de nous en parler.**

*Types de troubles (y compris les épilepsies) et les manifestations/symptômes rencontré(e)s le plus souvent chez les gens qui en souffrent*

*Comment voyez-vous les personnes souffrant d’un trouble de santé mentale ? Êtes-vous à l’aise en les rencontrant, en vivant avec elles ou éprouvez-vous les sentiments de peur, de tristesse ?*

*Quel regard la société a-t-elle sur ces personnes souffrant de troubles mentaux graves (c’est-à-dire celles qui ont perdu la raison et ne reconnaissent pas leurs maladies) ?*

**Parlez-nous comment les troubles de santé mentale constituent une charge sociale et de santé publique dans votre famille/zone de santé**

*Croyances des populations à l’égard de troubles*

*Intérêt de la population à sa santé mentale*

*Contribution à la mise en œuvre des activités de santé mentale, de soutien psychosocial et de pratiques de soins au niveau de la communauté*

**Connaissez-vous des personnes affectées par un problème/trouble de santé mentale dans votre famille/communauté ?**

*Rencontrez-vous quotidiennement des personnes en souffrance ?*

*Que faites-vous en les rencontrant : en famille, sur la rue… ?*

*Quelles catégories de personnes sont-elles beaucoup concernées par ces problèmes/troubles ?*

*Ces personnes sont-elles en errance dans la ville de Lubumbashi ? Adoptent-elles des comportements antisociaux ?*

*Ces personnes menacent-elles la tranquillité dans la famille/communauté ?*

*Quelles attitudes la communauté adopte-t-elle à l’égard de ces personnes : punitions, rejet, stigmatisation ? Merci de nous en parler.*

**1.2 Comportement de recherche de soins**

**Amenez les personnes avec troubles de santé mentale dans des services de soins de santé ou ailleurs pour qu’elles bénéficient d’une prise en charge ? Pourquoi/pourquoi pas ?**

*Direz-vous que c’est par charité que vous les amener aux soins ?*

*Direz-vous que c’est par devoir des parents/frères/sœurs que vous les amener aux soins ?*

**Recevez-vous des personnes avec troubles de santé mentale dans les services de soins de santé ?**

*Que faites-vous en les rencontrant : en famille, sur la rue, à l’hôpital… ?*

*Sensibilise-t-on la population sur les questions de santé mentale ? pourquoi ? par qui ?*

**Avant d’arriver au centre de santé_____ ou à la clinique, les personnes avec troubles de santé mentale vont-elles consulter ailleurs ? Merci de nous en dire plus.**

*Recours aux cabinets de médecine traditionnelle*

*Fréquentation des églises/groupes de prières/cabinets de consultation spirituelle*

*Fréquentation des chefs de familles et/ou chefs coutumiers*

THEME 2 : OFFRE DE SOINS DE SANTÉ MENTALE

**Pouvez-vous nous parler de la prise en charge des troubles de santé mentale au centre de santé de______ et aux cliniques privées ?**

*La prise en charge est-elle effective ?*

*Si oui, décrivez ce qui est réellement donné comme soins de santé mentale*

*Si non, quelles sont les raisons de la non-disponibilité de soins ?*

*Où sont orientés les malades qui ont besoin de soins de santé mentale ?*

*Existe-t-il des hôpitaux spécialisés dans votre zone de santé et/ou sur la ville de Lubumbashi qui offrent des soins de santé mentale ? Merci de nous en dire plus.*

*Quelles maladies soigne-t-on dans les hôpitaux psychiatriques ? À quel coût ?*

**Quels types de soins sont-ils actuellement possibles pour ces troubles dans votre communauté ?**

*Soins médicaux, avec médicaments psychotropes*

*Soins psychologiques : counseling, psychothérapies, médiation familiale, etc.*

*Soutien social aux personnes en difficultés*

*Prise en charge par des guérisseurs traditionnels*

*Séances des prières et d’exorcisme par des prêtres, des pasteurs,*

**Pouvez-vous nous parler de la façon dont les troubles de santé mentale sont pris en charge dans votre communauté ?**

*Dans les centres de santé – capacités des soignants à assurer cette prise en charge*

*Dans les hôpitaux – capacités des soignants à assurer cette prise en charge*

*Dans des structures de soins traditionnels – capacités des guérisseurs*

*Dans des familles et communautés – attitudes des proches aidants*

**Quel est le contenu du paquet minimum d’activités de santé des services de soins de santé primaires de votre zone de santé**

*Activités curatives*

*Activités préventives*

*Activités promotionnelles*

*Activités de réadaptation*

*Ce paquet d’activités intègre-t-il les soins de santé mentale ? Si oui, qu’est-ce qui est fait ? Si non, quelles sont les raisons ?*

**Pouvez-vous nous parler de suivi et orientation/référencement des cas de santé mentale ?**

*Qui effectue le suivi des cas de santé mentale ?*

*Une prise en charge financière est-elle proposée dans les services de soins de santé ?*

*Pouvez-vous orienter/référer les patients en cas de besoin ? Si oui, à qui ?*

THÈME 3 : CAPACITÉ DE RÉPONSE OPÉRATIONNELLE DE LA ZONE DE SANTÉ À RÉPONDRE AUX PROBLÈMES DE SANTÉ MENTALE

(Réservé à l’entretien avec les professionnels de santé)

**Pensez-vous que votre zone de santé a des capacités techniques requises pour lutter contre les troubles de santé mentale ?**

*Quels médicaments sont souvent disponibles : psychotropes, neuroleptiques y compris antiépileptiques*

*Circuit d’approvisionnement de ces médicaments*

**Pouvez-vous nous parler de la situation des médicaments psychotropes dans votre zone de santé (centre de santé_________, hôpitaux/cliniques, pharmacies ambulantes)**

*Quels médicaments sont souvent disponibles : psychotropes, neuroleptiques y compris antiépileptiques*

*Circuit d’approvisionnement de ces médicaments*

*Prix au niveau : a) de la centrale d’achat de la zone de santé, b) du centre de santé ____ et cliniques/centres hospitaliers, c) des pharmacies privées*

**Pouvez-vous nous parler des capacités des prestataires de soins à assurer la prise en charge des personnes avec des troubles de santé mentale ?**

*Leurs capacités à identifier, gérer les cas et assurer le suivi des patients*

*La formation dont ils ont bénéficié sur la santé mentale*

*Leurs capacités à utiliser le guide d’intervention combler les lacunes en santé mentale (mhGAP) de l’OMS si disponible dans les services de soins de santé*

**Pouvez-vous nous parler de la situation des médicaments psychotropes dans votre zone de santé (centre de santé_________, hôpitaux/cliniques, pharmacies ambulantes) ?**

*Quels médicaments sont souvent disponibles : psychotropes, neuroleptiques y compris antiépileptiques*

*Circuit d’approvisionnement de ces médicaments*

**Pouvez-vous nous parler de la façon dont la thématique de santé mentale est coordonnée dans votre zone de santé et dans la division provinciale du Haut-Katanga**

*Existence d’une coordination provinciale de santé mentale*

*Existence d’un superviseur en charge de santé mentale dans la zone de santé*

*Existence d’un référent santé mentale dans des centres de soins de santé primaires*

**Avez-vous quelque chose à ajouter concernant notre entretien ?**

**Quelles sont vos attentes par rapport à la thématique de santé mentale ?**

(Ces deux questions concernent tous les participants)
