# Supplementary material for: Mental health care in the city of Lubumbashi, Democratic Republic of the Congo: Analysis of demand, supply and operational response capacity of the health district of Tshamilemba
Source: PLoS One. 2023 Apr 5;18(4):e0280089. doi: 10.1371/journal.pone.0280089 (PMC10075459; doi:10.1371/journal.pone.0280089)
Supplement: S2 File — (DOCX) [file pone.0280089.s002.docx]

# Supplementary Text-File 2: Household Survey Questionnaire

**Household Survey Questionnaire**

(English version)

1. Information about the person collecting the data

1.1 Name of interviewer: ______________________ Phone number: +_ _ _ _ _ _ _ _ _ _ _ _

1.2 Date form completed (dd/mm/yyyy) ___/___/___
1.3 Date of respondent survey (dd/mm/yyyy) ___/___/___

2. Information about the respondent and his/her household

2.1 Unique ID: _____________ Health Area: ______________ Health Area: _______________

2.2 Gender Male □ Female □ Date of birth (dd/mm/yyyy) ___/___/___

2.3 Marital Status: Single □ Married □ Divorced □ Widowed □

2.4 Position in the household Head of household □ Spouse □ Elder in family □

2.5 Highest educational qualification obtained by the head of household

None □ Primary school certificate □ State diploma □

Graduate/university degree □ Other (specify) □

2.6 Sector of gainful activity of the head of household Civil servant □ Liberal □

If self-employed, specify activity _______________

2.7 Number of people in the household R/ _____ of which _____Children <5 years

3. Information on perceived/observed mental health problems

3.1 Have you experienced a psychosocial or mental health problem (a psychological problem or behavior that is considered abnormal in relation to community norms) in the past two weeks?

Yes □ No □ Don't remember □

3.2 Other than yourself, has anyone else in your household experienced a psychosocial or mental health problem in the past two weeks?

Yes □ No □ Don't remember □ If Yes🡪 How much? R/ _____

Note: If answer to Q3.2 is Yes🡪 skip Q3.3 and Q3.4, if No, stop here if Q3.1 is also No.

3.3 How old is he/she? A/ ___ ___ years (Note: If Q3.2 reveals more than one person, adapt Q3.3)

3.4 What gender is it? Male □ Female □

4. History of perceived/observed symptoms

Note: Complete this section only if Q3.1 and Q3.2 indicate the presence of a perceived or observed mental health problem.

4.1 During the past (X) weeks/months, have you or your family member experienced or observed any of the following signs and symptoms as reported in Q3.2?

| First group of signs/symptoms (PSY) | |
| --- | --- |
| Incoherent or irrelevant speech | □ Yes □ No □ Don't remember |
| Unreal ideas considered to be true (delusion) | □ Yes □ No □ Don't remember |
| Seeing and hearing things that do not exist (hallucinations) | □ Yes □ No □ Don't remember |
| Withdrawal | □ Yes □ No □ Don't remember |
| Agitation | □ Yes □ No □ Don't remember |
| Disorganized behavior | □ Yes □ No □ Don't remember |
| The impression that one's thoughts are stolen, guessed and imposed | □ Yes □ No □ Don't remember |
| A tendency to isolate oneself and to neglect one's usual tasks related to work, school, domestic or social activities | □ Yes □ No □ Don't remember |
| Lack of awareness that one has mental health problems | □ Yes □ No □ Don't remember |
| Second group of signs/symptoms (AUT) | |
| Multiple physical complaints that do not correspond to a known disease | □ Yes □ No □ Don't remember |
| Fear without apparent purpose | □ Yes □ No □ Don't remember |
| Nervousness (irritability) | □ Yes □ No □ Don't remember |
| Difficulty living or adapting in the community | □ Yes □ No □ Don't remember |
| Third group of signs/symptoms (DEP) | |
| Persistent sadness or depressed mood | □ Yes □ No □ Don't remember |
| Several persistent physical symptoms with no clear cause | □ Yes □ No □ Don't remember |
| Energy drop | □ Yes □ No □ Don't remember |
| Suicidal thoughts | □ Yes □ No □ Don't remember |
| Fatigue | □ Yes □ No □ Don't remember |
| Sleep problems | □ Yes □ No □ Don't remember |
| Anxiety | □ Yes □ No □ Don't remember |
| Loss of interest or pleasure in normally enjoyable activities | □ Yes □ No □ Don't remember |
| Fourth group of signs/symptoms (SUI) | |
| Extreme despair and hopelessness | □ Yes □ No □ Don't remember |
| Current thoughts, plan or act of self-harm/suicide, or history of same | □ Yes □ No □ Don't remember |
| Suicide attempts | □ Yes □ No □ Don't remember |
| Any other priority condition, chronic pain or extreme emotional distress | □ Yes □ No □ Don't remember |
| Fifth group of signs/symptoms (SUB) | |
| Person apparently under the influence of a psychoactive substance | □ Yes □ No □ Don't remember |
| Person showing a lack of energy | □ Yes □ No □ Don't remember |
| Agitation | □ Yes □ No □ Don't remember |
| Inability to sit still | □ Yes □ No □ Don't remember |
| Inarticulate language | □ Yes □ No □ Don't remember |
| Signs of substance use such as injection marks, skin infection, unkempt appearance | □ Yes □ No □ Don't remember |
| Person requesting a prescription for sedative medication (sleeping pills, opioids) | □ Yes □ No □ Don't remember |
| Person with financial difficulties or criminal problems | □ Yes □ No □ Don't remember |
| Person experiencing difficulties in performing usual work, domestic or social activities | □ Yes □ No □ Don't remember |
| Sixth group of signs/symptoms (ANX) | |
| Anxiety and worry associated with other symptoms | □ Yes □ No □ Don't remember |
| Restlessness or feeling of excitement or irritation | □ Yes □ No □ Don't remember |
| Extreme fatigue | □ Yes □ No □ Don't remember |
| Difficulty concentrating or a blank mind | □ Yes □ No □ Don't remember |
| Irritability | □ Yes □ No □ Don't remember |
| Muscle tension | □ Yes □ No □ Don't remember |
| Sleep disorders such as difficulty falling or staying asleep, or restless and unsatisfactory sleep | □ Yes □ No □ Don't remember |
| Seventh group of signs/symptoms (STR) | |
| Fear related to the stressful event | □ Yes □ No □ Don't remember |
| Feeling of powerlessness | □ Yes □ No □ Don't remember |
| Horror | □ Yes □ No □ Don't remember |
| Fear | □ Yes □ No □ Don't remember |
| Revival of traumatic memories (scenes, images) | □ Yes □ No □ Don't remember |
| Avoidance of traumatic memories and hyper vigilance, following an unfavorable or traumatic situation | □ Yes □ No □ Don't remember |

5. Views on the potential for the local health system to meet demands for care

In your opinion, are the health care services in your health district able to provide care for people with a mental health problem?

□ Yes □ No □ Don't know □

**Questionnaire d’enquête auprès des ménages**

(Version française)

1. Informations concernant la personne qui collecte les données

1.1 Nom de l’enquêteur : _______________ Numéro de téléphone portable : +_ _ _ _ _ _ _ _ _ _ _ _

1.2 Date de remplissage du formulaire (jj/mm/aaaa) ___/___/___
1.3 Date de l’enquête auprès du répondant (jj/mm/aaaa) ___/___/___

2. Informations concernant le répondant et son ménage

2.1 ID unique : _____________ Aire de santé : ______________ Zone de Santé : _______________

2.2 Sexe Masculin □ Féminin □ Date de naissance (jj/mm/aaaa) ___/___/___

2.3 État matrimonial : Célibataire □ Marié □ Divorcé □ Veuf(ve) □

2.4 Position dans le ménage Chef(fe) ménage □ Conjoint(e) □ Aîné de famille □

2.5 Titre scolaire le plus élevé obtenu par le chef de ménage

Aucun □ Certificat d’études primaires □ Diplôme d’État □

Diplôme d’études supérieures/universitaires □ Autre (préciser) □

2.6 Secteur d’activité lucrative du chef de ménage Fonctionnaire □ Libéral □

Si libéral, préciser l’activité _______________

2.7 Nombre des personnes qui composent le ménage R/ _____ dont _____Enfants <5 ans

3. Informations sur des troubles de santé mentale perçus/observés

3.1 Avez-vous été aux prises avec un problème psychosocial ou de santé mentale (problème psychique ou comportement jugé anormal par rapport aux normes du milieu) au cours de deux dernières semaines ?

Oui □ Non □ Ne se rappelle pas □

3.2 À part vous, y a-t-il quelqu’un d’autre au sein de votre ménage qui a présenté un problème psychosocial ou de santé mentale au cours de deux dernières semaines ?

Oui □ Non □ Ne se rappelle pas □ Si Oui 🡪 Combien ? R/ _____

Note : Si répondre au Q3.2 est Oui 🡪 passer Q3.3 et Q3.4, si Non, s’arrêter ici si Q3.1 est aussi Non.

3.3 Quel âge a-t-il ? R/ ___ ___ ans (Note : Si Q3.2 révèle plus d’une personne, adapter Q3.3)

3.4 De quel sexe est-il ? Masculin □ Féminin □

4. Historique des symptômes perçus/observés

Note : Remplir cette section seulement si Q3.1 et Q3.2 révèlent la présente d’un problème de santé mentale perçu ou observé.

4.1 Au cours des (X) dernier.e.s semaines/mois, avez-vous ressenti ou observé chez vous-même ou chez le membre de famille comme vous l’avez mentionné au Q3.2, certains d’entre les signes et symptômes suivants :

| Premier groupe de signes/symptômes (PSY) | |
| --- | --- |
| Discours incohérent ou non pertinent | □ Oui □ Non □ Ne se rappelle pas |
| Idées irréelles considérées comme vraies (délire) | □ Oui □ Non □ Ne se rappelle pas |
| Voir et entendre des choses inexistantes (hallucinations) | □ Oui □ Non □ Ne se rappelle pas |
| Retrait | □ Oui □ Non □ Ne se rappelle pas |
| Agitation | □ Oui □ Non □ Ne se rappelle pas |
| Comportement désorganisé | □ Oui □ Non □ Ne se rappelle pas |
| Impression que ses pensées sont volées, devinées et imposées | □ Oui □ Non □ Ne se rappelle pas |
| Tendance à s’isoler et à négliger ses tâches habituelles liées au travail, à l'école, aux activités domestiques ou sociales | □ Oui □ Non □ Ne se rappelle pas |
| Manque de prise de conscience que l'on a des problèmes de santé mentale | □ Oui □ Non □ Ne se rappelle pas |
| Deuxième groupe de signes/symptômes (AUT) | |
| Plaintes physiques multiples qui ne correspondent pas à une maladie connue | □ Oui □ Non □ Ne se rappelle pas |
| Peur sans objet apparent | □ Oui □ Non □ Ne se rappelle pas |
| Nervosité (irritabilité) | □ Oui □ Non □ Ne se rappelle pas |
| Difficultés de vivre ou de s’adapter en communauté | □ Oui □ Non □ Ne se rappelle pas |
| Troisième groupe de signes/symptômes (DEP) | |
| Tristesse persistante ou humeur dépressive | □ Oui □ Non □ Ne se rappelle pas |
| Plusieurs symptômes physiques persistants sans cause claire | □ Oui □ Non □ Ne se rappelle pas |
| Baisse d’énergie | □ Oui □ Non □ Ne se rappelle pas |
| Pensées suicidaires | □ Oui □ Non □ Ne se rappelle pas |
| Fatigue | □ Oui □ Non □ Ne se rappelle pas |
| Problèmes de sommeil | □ Oui □ Non □ Ne se rappelle pas |
| Anxiété | □ Oui □ Non □ Ne se rappelle pas |
| Perte d'intérêt ou de plaisir pour des activités normalement agréables | □ Oui □ Non □ Ne se rappelle pas |
| Quatrième groupe de signes/symptômes (SUI) | |
| Désespoir extrême et désespoir | □ Oui □ Non □ Ne se rappelle pas |
| Pensées actuelles, plan ou acte d’automutilation/suicide, ou antécédents de ceux-ci | □ Oui □ Non □ Ne se rappelle pas |
| Tentatives de suicide | □ Oui □ Non □ Ne se rappelle pas |
| Toute autre affection prioritaire, douleur chronique ou détresse émotionnelle extrême | □ Oui □ Non □ Ne se rappelle pas |
| Cinquième groupe de signes/symptômes (SUB) | |
| Personne apparemment sous l’effet d’une substance psychoactive | □ Oui □ Non □ Ne se rappelle pas |
| Personne manifestant un manque d’énergie | □ Oui □ Non □ Ne se rappelle pas |
| Agitation | □ Oui □ Non □ Ne se rappelle pas |
| Incapacité à se tenir tranquille | □ Oui □ Non □ Ne se rappelle pas |
| Langage inarticulé | □ Oui □ Non □ Ne se rappelle pas |
| Signes indiquant l’utilisation de substances psychoactives tels que marques d’injection, infection cutanée, apparence peu soignée | □ Oui □ Non □ Ne se rappelle pas |
| Personne formulant une demande de prescription de médicaments sédatifs (somnifères, opioïdes) | □ Oui □ Non □ Ne se rappelle pas |
| Personne ayant des difficultés financières ou problèmes criminels | □ Oui □ Non □ Ne se rappelle pas |
| Personne éprouvant des difficultés dans l’exécution des activités professionnelles, domestiques ou sociales habituelles | □ Oui □ Non □ Ne se rappelle pas |
| Sixième groupe de signes/symptômes (ANX) | |
| Anxiété et inquiétude associées à d’autres symptômes | □ Oui □ Non □ Ne se rappelle pas |
| Agitation ou sensation d'excitation ou d'énervement | □ Oui □ Non □ Ne se rappelle pas |
| Fatigue extrême | □ Oui □ Non □ Ne se rappelle pas |
| Difficulté à se concentrer ou esprit vide | □ Oui □ Non □ Ne se rappelle pas |
| Irritabilité | □ Oui □ Non □ Ne se rappelle pas |
| Tension musculaire | □ Oui □ Non □ Ne se rappelle pas |
| Troubles du sommeil tels que difficulté à s'endormir ou à rester endormi, ou sommeil agité et insatisfaisant | □ Oui □ Non □ Ne se rappelle pas |
| Septième groupe de signes/symptômes (STR) | |
| Peur liée à l’évènement stressant | □ Oui □ Non □ Ne se rappelle pas |
| Sentiment d’impuissance | □ Oui □ Non □ Ne se rappelle pas |
| Horreur | □ Oui □ Non □ Ne se rappelle pas |
| Effroi | □ Oui □ Non □ Ne se rappelle pas |
| Reviviscence des souvenirs (scènes, images) traumatisants | □ Oui □ Non □ Ne se rappelle pas |
| Évitement des souvenirs traumatiques et hyper vigilance, suite à une situation défavorable ou traumatisante | □ Oui □ Non □ Ne se rappelle pas |

5. Opinions sur les possibilités pour le système de santé local à répondre aux demandes de soins

À votre avis, les services de soins de votre zone de santé sont-ils en mesure d’offrir les soins aux personnes affectées par un problème de santé mentale ?

□ Oui □ Non □ Ne sait pas □
