# Supplementary material for: Mental health care in the city of Lubumbashi, Democratic Republic of the Congo: Analysis of demand, supply and operational response capacity of the health district of Tshamilemba
Source: PLoS One. 2023 Apr 5;18(4):e0280089. doi: 10.1371/journal.pone.0280089 (PMC10075459; doi:10.1371/journal.pone.0280089)
Supplement: Attachment — Submitted filename: Response to Reviewers.docx [file pone.0280089.s004.docx]

Erick **Mukala Mayoyo**, PhD student

School of Public Health, University of Lubumbashi, DR Congo

School of Public Health, Université Libre de Bruxelles, Belgium

E: [erickmukala1@gmail.com](mailto:erickmukala1@gmail.com) / M: +243 (0) 817 292 399

Kasapa Street, commune of Lubumbashi, DR Congo

Lubumbashi, February 28, 2023

To the Academic Editor of PLOS ONE journal

Subject: **Response to Reviewers** about the manuscript–PONE-D-22-34666 ‘Mental health care in the city of Lubumbashi, Democratic Republic of the Congo: Analysis of demand, supply and operational response capacity of the health district of Tshamilemba’

Dear Madam, Sir,

While thanking, through you, the reviewers and editorial team for their valuable work, I am pleased to submit for publication our revised manuscript PONE-D-22-34666, based on the comments and suggestions that were formulated.

The following changes [red text] were necessary, which we have made in the revised manuscript in blue text:

**The requested journal requirements were:**

1. [Ensure that the manuscript meets PLOS ONE's style requirements]. We did so.

2. [Provide copies of the interview guide and survey as supplementary files]. We provided the requested files.

3. [Provide the correct grant numbers for the awards you received for your study in the ‘Funding Information’ section]. We provided the grant number in ‘finding information’ section (number: 901004/470).

4. [Review your reference list to ensure that it is complete and correct]. We have made some changes to the reference list. Reference [29] in the unrevised manuscript was directly cited in the text, as it is part of the grey literature available only at the health district office. Reference [39] has been deleted within the corresponding text, as per one reviewer’s request. In addition, 10 new references [22, 31, 33, 34, 36, 40, 48, 49, 51, 52] have been added to the revised manuscript.

We added some information in the manuscript to fully adhere to the COREQ and STROBE guidelines. For the COREQ guidelines, the clarifications provided relate to the following: research team; study design (methodological orientation, sampling, method of approach, data collection); and analysis and findings. Moreover, for the STROBE directives, the clarifications provided concern the abstract; methods (study design, statistical methods), results (characteristics of study participants); and discussion (interpretation) of the findings. The changes made are on the following lines: 205; 241; 280; 282; 287-289; 290; 326-330; 349; 353-354; 393-396, in the revised manuscript.

**Point by point responses to reviewers’ comments:**

Note: Line numbers in brackets refer to the revised manuscript to indicate the changes made.

**Abstract:**

Methods

[What type of survey was conducted?]. We specified: a ‘household survey’. (Line 36)

[It is not clear how the burden of mental health problems was assessed, nor how demand was analysed, or how the district’s operational capacity was assessed]. We specified as follows: ‘The burden of mental disorders was assessed by calculating a morbidity indicator (proportion of mental health cases) and through a qualitative analysis of the psychosocial consequences as perceived by the participants. Care-seeking behavior was analyzed by calculating health service utilization indicators and more specifically the relative frequency of mental health complaints in primary health care centers, and by analyzing FGDs participants’ reports’. The mental health care supply available was described ‘by using the qualitative analysis of the declarations of the participants (providers and users of care) to the FGDs and by analyzing the package of care available in the primary health care centers’. Finally, the district’s operational response capacity was assessed ‘by making an inventory of all available resources and by analyzing qualitative data provided by health providers and managers regarding the district’ capacity to address mental health conditions’. (Line 40 to 51)

Results
[Which methods led to the conclusion that traditional medicine is the main source of care?]. We specified as follows: ‘Participants in the FGDs stated that in this context, the main source of care for people remains traditional medicine’. (Line 60)

Conclusion
[While I agree that integrating a basic package of mental health care into primary care seems to be relevant, it is not clear how the results support such a specific intervention. Mental health care seems to be lacking at all levels of the health care system and this study identified multiple barriers to access. Moreover, this study was conducted in one small health district, and one should be careful to generalize the findings to the entire country, or at least argue why the authors think the situation is similar in most areas]. We reformulated the conclusion as follows: ‘Our findings show a clear demand for mental health care and a lack of formal mental health care supply in the Tshamilemba district. Moreover, this district lacks adequate operational capacity to meet the mental health needs of the population. Traditional African medicine is currently the main source of mental health care in this health district. Identifying concrete priority mental health actions to address this gap, by making evidence-based mental care available, is therefore of great relevance.’ (Line 64 to 69)

**Introduction**

[Beds in outpatient facilities seem contradictory. Maybe inpatient facilities?]. Indeed, these are ‘inpatient facilities’. We corrected. (Line 94)

[Not clear. Earlier you mention that mental health care has been integrated in Guinea, and here you state that it is not widely integrated]. In Guinea, integration was achieved in only five health centers, all members of an NGO-driven mental health programme. We specified this in the revised manuscript. In this sentence, we also specified that in most LMICs there is no offer of mental health at primary care level. The sentence becomes: However, the fact that the mental health care is not integrated into PHC services ‘in most LMICs’ makes the coverage of mental health services very low. (Line 106)

[It seems you are conflating two issues into one: insufficient financing and lack of integration at primary care]. We separated the two issues ‘insufficient financing and lack of integration at primary care’. The two currently separate issues ‘an insufficient annual national budget allocated to the mental health subsector’ and ‘lack of integration of mental health provision into primary care in most LMICs’ (Line 87 to 89)

[Use of traditional medicine and other alternative approaches in a situation where no other mental health care is available seems a solution, albeit imperfect, rather than a problem]. We agree. We have indicated in the revised manuscript that the use of traditional medicine and other healing practices is an alternative and not a problem. The sentence becomes: Third, due to a lack of formal mental health provision, patients resort to ‘alternative solutions such as’ traditional medicine, herbal medicine and quackery… (Line 108 to 109)

[What does this DALY refer to? The burden of all mental health disorders in DRC or the burden due to suicide? How does this compare with other countries?]. The DALY refer to mental and substance use disorders. We specified in the revised manuscript. We also compared with other countries. The sentence becomes: Based on 2020 World Health Organization (WHO) official estimations on the burden of mental disorders, in the DRC, the DALY (per 100 000 population) ‘for mental and substance use disorders’ was 1 557.7 ‘compared to other countries in the region such as Cameroon, Central African Republic, Congo and Gabon where the DALYs were respectively 347.7, 88.4, 85.7 and 33.7 [21,22]’… (Line 127 to 130)

**Methods**

[Do these numbers refer to the health district of Tshamilemba or Lubumbashi? If Tshamilemba, does this mean there is one public PHC for 283 000 inhabitants? And 59 private clinics? If not, what are the numbers for Tshamilemba?]. Exactly. There is only one public primary care facility in Tshamilemba district, called Center de Santé de Tshamilemba. The other 59 health services are private clinics. We reworded the sentence as follows: ‘The 2020 consolidated operational action plan of the health district of Tshamilemba indicates that this district, which covers an area of about 42 km^2^ and a population of 283 000 people, is divided into 13 health areas; counts 59 private clinics and only one public primary care facility, i.e. Tshamilemba PHC center, covering about 12 000 people. This clearly illustrates the huge (and problematic) extent of privatization of the first level of health care in the city of Lubumbashi in general, and in the district of Tshamilemba in particular. (Line 182 to 189)

[In line 140 it is stated 10 000 population]. The 10,000 inhabitants in line 140 was a multiplier of the mental health human resources availability indicator and not the number of inhabitants of the Tshamilemba district. For the sake of clarity, we have corrected this by referring to the entire population of the district (283 000 inhabitants) and not to the population of the health area where the Tshamilemba PHC center is based (12 334 inhabitants). (Line 153)

[It is not clear what this formula refers to and to measure which effect the sample size was calculated. Also, a reference is needed]. We have added some information to make the formula used understandable. We also added a reference. The sentence becomes: The sample size was calculated for the two clusters at 769 subjects by applying the following formula [31]: $n=Za^{2}*c*(pq)/(d^{2})$, where : $Za^{2} = 1.96^{2}$; ‘$c=2$(number 2, means clusters factor used to minimize the effect of assumed heterogeneity of the study area)’; $p = 0.5$ ‘and’ $q = 0.5$ ‘(as the prevalence of mental health problems is not known in the study setting)’ and $d^{2} = 0.05^{2}$. (Line 297 to 300)

[These numbers should be in the Results section]. We brought them back in the results section. (Line 391 to 396)

[Survey questionnaires and focus group discussion guides should be made available in supplementary files and referenced]. We made them available in supplementary files.

**Results**

[Unclear. Is this the proportion of all general consultations that was for mental health care?]. This is the proportion of mental health cases among all general consultations carried out. We have reformulated the sentence as follows to make it more clear: From these results, we note that in 2020, of all general curative consultations carried out at the Tshamilemba primary care center, the proportion of patients with mental health problems out of all curative consultations was 5.3%. (Line 416 to 419)

[Table 3. Population is stated to be 10,000 and also 12,000 above. Need to harmonize numbers. Thanks a lot. Normally, the population of responsibility of the Tshamilemba PHC center is 12 000 (11 975) inhabitants. We harmonized. (Line 423)

What does (=2/1; 4/2; 4/1) refer to; need to clarify in the legend. In general, this table would be clearer if you specify the numerator and the denominator]. =2/1; 4/2; 4/1 were formulas. For example, in 2/1, 2 was the numerator and 1 the denominator. We have placed this in the legend with superscripts 1, 2, and 3 and explanations for clarity. The added explanations are:

^1^Numerator: Number of new cases (NCs) at the curative consultation at the Tshamilemba PHC center in 2020; Denominator: Population of the health subdistrict of Tshamilemba (estimated in 2020) (n=11 975).

^2^Numerator: Number of NCs with mental health problems seen at the curative general consultation at the Tshamilemba PHC center; Denominator: total number of NCs at the curative consultation at the Tshamilemba PHC center in 2020 (n=3 041).

^3^Numerator: Number of NCs with mental health problems seen at the curative general consultation at the Tshamilemba PHC center; Denominator: Population of the health subdistrict of Tshamilemba (estimated in 2020) (n=11 975). (Line 423)

[Specify numerator/denominator]. We specified the numerator and denominator. The sentence then becomes: Out of a total of 3 041 patients seen at the Tshamilemba PHC center in 2020, there were 40 cases of epilepsy, representing a proportion of 1.3%. (Lines 426 to 427)

[Table 4. Title needs to specify that these are results from the household survey. We agree! The title becomes: Table 4. Distribution of mental health cases reported by respondents in the household survey (n = 591). (Lines 456 to 457) The age comparison is not particularly useful. I would suggest removing it from the table and only referring to overall mean difference in the text]. We removed age comparison from the table. Instead of referring to the overall mean difference, we simply referred to the fluctuations of the sample statistic, i.e. the proportion, with the according 95% confidence interval. (Lines 460 to 464)

[Table 4. Which definitions did you use for the mental health problems categories? For example, what is the difference between stress & anxiety problems? How did you define neuroses and psychoses?]. We paraphrased definitions presented in the compendium of the National Mental Health Program of the DRC, which referred to the WHO mhGAP-IG (mental health Gap Action Program-Intervention Guides). We added in the method section a sub-section related to "**Definitions of main mental health disorders categories used**" with content as presented below: (Lines 313 to 320)

In this study, we used the definitions presented by the National Mental Health Program of the DRC in its mental health module of the NHIS (National Health Information System). In this module, the Program refers to the WHO mental health Gap Action Program-Intervention Guides (mhGAP-IG). The symptomatic approach has been adopted to clearly define these concepts, in order to facilitate their understanding by all non-specialists (i.e. community health workers, family members, primary care providers) participating in the study. These definitions are presented in Box 1 in the revised manuscript (Lines 319-320) as follows:

**Box 1. Definitions of main mental health disorders**

**Anxiety disorders:** in the past 6 months, regular (i.e. more often than not) experiencing disorders in which anxiety and concerns are associated with at least three of the following six symptoms: restlessness or feelings of excitation or nervousness, fatigue, difficulties in concentration, irritability, muscular tension, sleeping problems and restlessness. **Stress disorders:** patients experience fear when facing stressful events, feeling of helplessness, horror, fright, reliving traumatic memories (scenes, images); avoidance of traumatic memories and hypervigilance following adverse or traumatic situations. **Depression:** A disorder in which the patient experiences persistent sadness or depressed mood, fatigue, sleep problems, decreased energy and suicidal thoughts, anxiety, loss of interest or pleasure in normally enjoyable activities for at least 2 weeks. **Substance Use and Associated Disorders:** Disorders occurring in a person apparently under the influence of a psychoactive substance, manifested by, for example, lack of energy, agitation, inability to sit still, inarticulate speech; or signs indicative of substance use such as injection marks, skin infection, disordered appearance; requesting a prescription for sedative medication (sleeping pills, opioids); having financial difficulties or criminal problems; or difficulty with work, at household level, or in habitual social activities. **Suicidal Thoughts and Attempts:** Disorders in which patients experience extreme hopelessness and despair; with (past or present) thoughts, plans, or acts of self-mutilation, suicide attempts, and any other condition associated with chronic pain or extreme emotional distress. **Neuroses:** Disorders in which patients experience multiple physical complaints, without apparent medical explanation or physical cause, and that do not correspond to a known illness; experience of fear without apparent reason, nervousness, difficulties in living in community. **Psychoses:** Disorders in which patients exhibit incoherent or irrelevant speech; delusions (unreal ideas believed to be true); hallucinations (hearing voices and seeing things that do not exist); feelings of withdrawal, restlessness, disorganized and aggressive behavior, fixation on false beliefs not shared by others in the person's culture, feeling that one's thoughts are being stolen, or imposed; tendency to isolate oneself and neglect habitual tasks and responsibilities related to work, school, domestic or social activities, with lack of awareness that one has mental health problems.

[Young age was (NOT has been shown to be) a protective factor. In general, young age is not protective for substance use disorder and depression. It may be an interesting question to explore what this would be the case in this district of DRC]. As the reviewers advised us to drop the comparison of average ages among people with mental health disorders, this finding was removed from the result section. But, we included this question in the conclusion section to be explored in our future research. We wrote it thus at the end of the conclusion:

Our findings showed that young people suffered less than adults from substance use disorders and depression. Yet we generally know that young age is not a protective factor against these disorders. There is a need to explore why this would be the case in this district of DRC. (Lines 781 to 784)

[The data you describe on epilepsy are very interesting and reflect the importance of epilepsy as a major neglected neurological condition, with high stigma and grossly inadequate management. The fact that it is considered a mental health condition and inadequately treated with diazepam and promethazine is telling]. This is a positive point. We kept it as such. (Lines 492 to 494)

[The available treatments for psychiatric illnesses also seem to be severely lacking]. We agree. We added it, and the sentence becomes: In addition, they have noticed that past attempts to treat patients with other mental health disorders (depression, psychoses…) by administering psychotropic drugs (amitriptyline, chlorpromazine) often failed, with patients returning to their family and community with unmet needs, showcasing that treatments for psychiatric illnesses are grossly lacking. (Line 497 to 498)

[39000 USD seems an extremely low amount for a country of 90 million inhabitants. Can you reference the source and also add the total national budget and the total amount allocated to health care? This would add some necessary perspective]. Point taken. We reformulated the sentence by referencing the information source and also adding the total national budget and the total amount of it allocated to health care. The sentence then becomes: Analysis of the country’s 2022 finance bill indicates that the overall national budget was US$9.9 billion (US$1 = CDF 2 093). Yet, the health sector budget forecast documents we analyzed revealed that the share of the national budget allocated to the health sector in 2022 was US$225 million. Of this amount, the share specifically allocated to mental health care was US$39 000. (Line 572 to 576)

**Discussion**

[Some general comments on the discussion. It would benefit from highlighting the results more and placing them in the context of existing evidence. Topics on which presenting existing evidence would be useful include mental health burden of disease and needs, mental health care offer in DRC and Africa, traditional healers as mental health providers, burden and care of epilepsy, access to medication for psychiatric disorders and epilepsy, access to counselling and psychotherapy. Your findings highlight significant stigma, both from providers and from the general public, surrounding mental health. This should also be addressed in the discussion].

We tried to better highlight the results and place them in the context of the existing evidence. We added information on the following topics: burden of disease and mental health needs, provision of mental health care, traditional healers as providers of mental health care, burden and epilepsy care, access to medication for psychiatric disorders and epilepsy, access to counseling and psychotherapy. We also added information about mental health stigma. This extra information is referenced by the appropriate sources.

Regarding the burden of disease, the second paragraph has been revised as follows:

This study highlights that the burden of mental health problems is significant in this urban area. (Line 594). In the household survey, half of the respondents reported mental health problems either themselves or in their family. Three disorders were more common: stress disorders, substance use disorders, and depression. The literature does not indicate that young age is protective against substance use disorders and depression. However, the findings seem to show that young people suffered less than adults from these two disorders. There is a need to explore why this would be the case in this district of DRC. Documentary analysis showed that in 2020, 5.3% of all general consultations in PHC centers in Tshamilemba concerned mental health. According to the 2019 Global Burden of Disease study [33], the prevalence of mental disorders including substance use disorders in the DRC is estimated at 13.23% of the population, i.e. 11.42 million people. This prevalence is likely to be underestimated because except for the provincial health divisions of North Kivu and South Kivu which are currently connected to the District Health Information Software 2 (DHIS2), in the other provincial health divisions, mental health data is not routinely notified across primary care health services. Moreover, the epidemiological burden would probably be much heavier if population surveys were carried out, because the people in Congo generally do not consult for minor mental health conditions. There are currently no specific survey data available to determine the real extent of the situation. The present research precisely aims to fill this gap. (Line 595 to 605)

Regarding mental health care provision, we added the following paragraph:

The results indicated that only 1.4% of people with mental disorders had access to curative mental health care; thus sparking the debate on the very poor availability and accessibility of mental health care in the DRC. Based on this result, we can reasonably well assume that there is a significant mental health treatment gap in the Tshamilemba district, certainly when compared to the general figure of 10% of people with mental disorders accessing evidence-based treatments in LMICs overall [34]. (Line 609 to 617)

Regarding the burden and epilepsy care, revised the fifth paragraph by adding the following:

Furthermore, the findings pointed to the fact that epilepsy is currently not properly taken care of in the Tshamilemba district. Indeed, no effective anti-seizure medicines are available besides diazepam, which is used only for acute cases. The unmet need of epilepsy treatment is therefore considerable. The majority of people living with this neurological condition can unfortunately not access proper care. As in most LMICs where the treatment gap exceeds 75% [36], there is need to integrate epilepsy care in existing primary health care services. (Line 626 to 632)

Regarding the traditional healers as providers of mental health care, we added the following paragraph:

According to the beliefs of the inhabitants of Lubumbashi, traditional African medicine remains the main recourse for treating mentally ill people. Research indicated that in the DRC, the first contact of mentally ill people with a health care provider is with traditional healers and/or spiritual workers (priests, lay pastors or imams), and that only as last resort modern health professionals such as psychiatrists and psychologists are contacted [40]. Also in the Tshamilemba district, traditional healers are the main source of care for mental health problems; future research into the effectiveness of their services remains however to be done. (Line 646 to 652)

We added the following paragraph about access to medication for psychiatric disorders and epilepsy:

To date, access to medication for psychiatric disorders and epilepsy is very limited in the health district of Tshamilemba. According to a 2022 country analysis [48], the lack of availability of essential antipsychotic and neuroleptic drugs, combined with their prohibitive cost, contributes to further worsen the treatment adherence. (Line 706 to 709)

We added the following paragraph about access to counseling and psychotherapy:

Our findings revealed that access to counseling and psychotherapy was virtually non-existing in Tshamilemba district. These results corroborate those of the 2022 country analysis which affirmed that the possibilities of counselling and psychotherapy in the DRC are extremely poor [48]. However, some local or international non-profit organizations provide some level of support services for victims to sexual violence. Additionally, mental health and psychosocial support is a growing concern of humanitarian organizations, especially in conflict and post-conflict areas. (Line 710 to 716)

We also added the following paragraph about mental health stigma:

Stigmatizing attitudes vis-à-vis mental health problems are unfortunately (still) quite dominant in the community. These attitudes are related to the prevailing belief that mental disorders are linked to supernatural causes [20] such as witchcraft, sorcery, magic, bad spirits, bewitchment, transgression of taboos… These beliefs are largely shared by family members of people with mental health problems. Primary care providers acknowledge their limitations on how to address mental health problems. This contributes to leading people facing mental health problems to seek help from traditional healers and/or spiritual workers [49]. (Line 717 to 724)

[This urban area. The study only provides data on one area]. We agree. We specified: …is significant in this urban area. (Line 594)

[I would be very cautious to suggest specific causes such as coronaphobia given that the study did not uncover any detailed diagnostic data and that the diagnostic term of neurosis is outdated and rather vague in this context. Rather highlight the need for further research to characterize the burden of mental health in DRC]. We agree. We reworded the whole paragraph which now becomes as written below:

Surveyed participants stated that they and their family members were mostly affected by stress-related disorders, substance abuse and depression. There is however need for a more thorough investigation of the precise nature of the burden of mental health in the DRC in general, and in Tshamilemba district in particular. (Line 608 to 611)

[I agree it is very interesting, although unsurprising, that epilepsy is considered a mental health disorder. I would highlight that at the moment epilepsy is not managed appropriately as according to the results there were no proper antiseizure medications available besides diazepam, which is used only in for acute seizures. Maybe it is also worth highlighting the enormous unmet need of epilepsy treatment worldwide, with more than 70% of people living with epilepsy not accessing care1]. We already explained this on the previous page 6, third paragraph regarding the burden and epilepsy care, and fifth paragraph related to access to medication for psychiatric disorders and epilepsy.

[Remove with]. (The findings showed that patients, including those who are mentally ill, who were followed up with at the Tshamilemba PHC center paid for their care out-of-pocket because they were not covered by the social protection system) We removed the paragraph.

[Title should be Strengths and Limitations. Please also highlight strengths of your study]. We changed the title to ‘Strengths and limitations’, and added the two following strengths:

A first strength of our study is that, to the best of our knowledge, it is the first ever conducted in the city of Lubumbashi - and perhaps in the whole of the DRC - to analyze the demand and supply of mental health care, as well as the operational capacity of the health districts to address the huge unmet mental health needs of people. Our findings may inspire researchers, policy makers and health managers and constitute a basis for further reflection on the development of more evidence-based strategies to improve access to quality mental health care at the primary level.

A second strength of our study lies in its methodological set-up. The multimethod approach used, with triangulation of sources, methods and data, contributes to enhance the validity of our findings. (Line 726 to 736)

**Conclusions**

[While I agree that a pilot project would a valuable response to the unmet needs in mental healthcare described in the study, this article is not the place to provide a detailed plan for such a project. Conclusions should be based on the results and propose future directions for research and public health action. I would suggest to shorten the conclusions, base them more closely on the results, and add additional references on the proposed responses].

We agree. We shortened the conclusions, and tried to ground the findings and suggest some future directions for public health action. We added two additional references [51, 52] on the proposed responses. The ‘conclusion’ section currently looks as follows:

Our study established that the demand for mental health care in the Tshamilemba urban district in the city of Lubumbashi increased over time, but that, at the same time, the formal supply of mental health care remains poor. In Tshamilemba, traditional medicine remains the main source of mental health care provision. The current lack of evidence-based treatment results from a combination of poor operational capacity of primary care facilities, lack of resources, low priority of mental health care at policy level, and, last but not least, the reluctance of people with mental health problems to seek care given the high level of stigmatization of mental health issues.

The unmet mental health care needs are significant, and the responses to address them are grossly insufficient and inadequate. The need for urgent action in the area of mental health in this urban district, and undoubtedly throughout the city of Lubumbashi, is clear indeed. Among the 12 key shifts recommended by the WHO [51] to transform the mental health situation, three seem particularly relevant for our study setting: first, invest in universal health coverage policies that explicitly incorporate mental health care; second, raise community awareness for mental health problems and fight stigmatization and discrimination; and third, integrate a basic package of mental health care into existing primary care services. As Alegría et al. [52] point out rightly, such an ambition endeavor requires investing in the health workforce through task‐shifting, training and supervision of primary care providers, delivering care close to where people live rather than expecting them to travel long distances to access residential psychiatric care. Our findings showed that young people suffered less than adults from substance use disorders and depression. Yet we generally know that young age is not a protective factor against these disorders. There is a need to explore why this would be the case in this district of DRC. (Lines 762 to 784)

Looking forward to seeing our manuscript published in the coming weeks, please accept, Madam, Sir, the expression of my most distinguished consideration.

Kind regards,

Erick Mukala Mayoyo
